# Supplementary material for: Modeling Within-Host Dynamics of SARS-CoV-2 Infection: A Case Study in Ferrets
Source: Viruses. 2021 Aug 18;13(8):1635. doi: 10.3390/v13081635 (PMC8402735; doi:10.3390/v13081635)
Supplement: Supplementary file 1 [file viruses-13-01635-s001.zip › viruses-1288793-supplementary.pdf]

Table S1. Pearson Correlation Coefficient of Estimated Parameters from Replicates by Bootstrapping the Residuals.

Model 1

Animal F13-E-1

	β	δ	α	p	c	$V_{i(}$
β	1.00	-0.06	0.09	-0.11	0.16	0.03
δ	-0.06	1.00	-0.37	0.35	-0.50	-0.30
α	0.09	-0.37	1.00	-0.30	0.74	0.44
p	-0.11	0.35	-0.30	1.00	-0.04	-0.31
c	0.16	-0.50	0.74	-0.04	1.00	0.40
$V_{i(}$	0.03	-0.30	0.44	-0.31	0.40	1.00

Animal F13-E-2

	β	δ	α	p	c	$V_{i(}$
β	1.00	-0.21	0.04	-0.34	0.00	-0.01
δ	-0.21	1.00	-0.38	0.36	-0.47	-0.15
α	0.04	-0.38	1.00	-0.33	0.68	0.12
p	-0.34	0.36	-0.33	1.00	0.04	-0.12
c	0.00	-0.47	0.68	0.04	1.00	0.14
$V_{i(}$	-0.01	-0.15	0.12	-0.12	0.14	1.00

Animal F13-E-3

	β	δ	α	p	c	$V_{i(}$
β	1.00	-0.04	-0.14	0.03	0.01	-0.08
δ	-0.04	1.00	-0.31	0.27	-0.45	0.07
α	-0.14	-0.31	1.00	-0.22	0.51	-0.05
p	0.03	0.27	-0.22	1.00	0.15	-0.15
c	0.01	-0.45	0.51	0.15	1.00	0.03
$V_{i(}$	-0.08	0.07	-0.05	-0.15	0.03	1.00

Animal Ctan-H-1

	β	δ	α	p	c	$V_{i(}$
β	1.00	0.07	0.01	0.05	0.22	0.12
δ	0.07	1.00	-0.29	0.20	-0.46	-0.30
α	0.01	-0.29	1.00	-0.20	0.58	0.34
p	0.05	0.20	-0.20	1.00	0.04	-0.29
c	0.22	-0.46	0.58	0.04	1.00	0.32
$V_{i(}$	0.12	-0.30	0.34	-0.29	0.32	1.00

Animal Ctan-H-2

	β	δ	α	p	c	$V_{\text{i(}}$
β	1.00	0.09	-0.24	0.01	-0.19	-0.01
δ	0.09	1.00	-0.40	0.54	-0.49	-0.05
α	-0.24	-0.40	1.00	-0.30	0.60	-0.07
p	0.01	0.54	-0.30	1.00	0.05	-0.03
c	-0.19	-0.49	0.60	0.05	1.00	0.08
$V_{\text{i(}}$	-0.01	-0.05	-0.07	-0.03	0.08	1.00

Animal Ctan-H-3

	β	δ	α	p	c	$V_{\text{i(}}$
β	1.00	0.29	-0.51	0.36	-0.18	0.09
δ	0.29	1.00	-0.38	0.28	-0.49	-0.02
α	-0.51	-0.38	1.00	-0.29	0.53	-0.06
p	0.36	0.28	-0.29	1.00	0.11	-0.03
c	-0.18	-0.49	0.53	0.11	1.00	-0.03
$V_{\text{i(}}$	0.09	-0.02	-0.06	-0.03	-0.03	1.00

Model 2

Animal F13-E-1

	β	δ	α	p	c	k	$V_{\text{i(}}$
β	1.00	-0.22	-0.48	-0.02	-0.03	0.30	-0.19
δ	-0.22	1.00	0.20	-0.08	-0.32	-0.36	0.14
α	-0.48	0.20	1.00	-0.19	0.16	-0.04	0.20
p	-0.02	-0.08	-0.19	1.00	0.07	-0.15	-0.11
c	-0.03	-0.32	0.16	0.07	1.00	0.20	0.13
k	0.30	-0.36	-0.04	-0.15	0.20	1.00	-0.03
$V_{\text{i(}}$	-0.19	0.14	0.20	-0.11	0.13	-0.03	1.00

Animal F13-E-2

	β	δ	α	p	c	k	$V_{\text{i(}}$
β	1.00	-0.18	-0.49	-0.17	-0.09	-0.10	-0.18
δ	-0.18	1.00	0.24	0.01	-0.32	-0.17	-0.03
α	-0.49	0.24	1.00	-0.16	0.14	0.16	0.06
p	-0.17	0.01	-0.16	1.00	0.12	0.07	-0.10
c	-0.09	-0.32	0.14	0.12	1.00	0.29	0.13
k	-0.10	-0.17	0.16	0.07	0.29	1.00	-0.02
$V_{\text{i(}}$	-0.18	-0.03	0.06	-0.10	0.13	-0.02	1.00

Animal F13-E-3

	β	δ	α	p	c	k	$V_{i(}$
β	1.00	-0.24	-0.52	0.06	0.12	-0.11	-0.31
δ	-0.24	1.00	0.17	-0.09	-0.42	0.28	0.13
α	-0.52	0.17	1.00	-0.27	0.06	0.09	0.10
p	0.06	-0.09	-0.27	1.00	0.21	0.11	-0.14
c	0.12	-0.42	0.06	0.21	1.00	0.11	-0.06
k	-0.11	0.28	0.09	0.11	0.11	1.00	0.12
$V_{i(}$	-0.31	0.13	0.10	-0.14	-0.06	0.12	1.00

Animal CTan-H-1

	β	δ	α	p	c	k	$V_{i(}$
β	1.00	-0.04	-0.24	-0.10	0.17	-0.15	-0.15
δ	-0.04	1.00	0.10	-0.06	-0.15	0.07	0.17
α	-0.24	0.10	1.00	-0.07	-0.06	0.03	0.43
p	-0.10	-0.06	-0.07	1.00	0.02	0.02	-0.03
c	0.17	-0.15	-0.06	0.02	1.00	-0.02	-0.03
k	-0.15	0.07	0.03	0.02	-0.02	1.00	0.34
$V_{i(}$	-0.15	0.17	0.43	-0.03	-0.03	0.34	1.00

Animal CTan-H-2

	β	δ	α	p	c	k	$V_{i(}$
β	1.00	-0.30	0.19	-0.14	0.12	-0.30	-0.26
δ	-0.30	1.00	0.18	-0.17	-0.42	0.44	0.28
α	0.19	0.18	1.00	-0.16	-0.11	0.13	0.16
p	-0.14	-0.17	-0.16	1.00	0.28	0.04	-0.15
c	0.12	-0.42	-0.11	0.28	1.00	-0.03	-0.10
k	-0.30	0.44	0.13	0.04	-0.03	1.00	0.36
$V_{i(}$	-0.26	0.28	0.16	-0.15	-0.10	0.36	1.00

Animal CTan-H-3

	β	δ	α	p	c	k	$V_{i(}$
β	1.00	-0.04	-0.53	0.11	0.12	0.24	-0.22
δ	-0.04	1.00	0.02	0.01	-0.36	-0.14	0.28
α	-0.53	0.02	1.00	-0.07	0.31	-0.28	0.09
p	0.11	0.01	-0.07	1.00	0.38	0.06	-0.09
c	0.12	-0.36	0.31	0.38	1.00	0.16	-0.04
k	0.24	-0.14	-0.28	0.06	0.16	1.00	0.05
$V_{i(}$	-0.22	0.28	0.09	-0.09	-0.04	0.05	1.00

Model 3

Animal F13-E-1

	β	δ	α	p	c	σ	$V_{i(}$
β	1.00	-0.28	-0.45	0.00	0.27	-0.15	-0.25
δ	-0.28	1.00	0.24	-0.05	-0.28	-0.04	0.08
α	-0.45	0.24	1.00	-0.13	0.15	0.09	0.09
p	0.00	-0.05	-0.13	1.00	-0.02	-0.06	-0.10
c	0.27	-0.28	0.15	-0.02	1.00	-0.19	-0.04
σ	-0.15	-0.04	0.09	-0.06	-0.19	1.00	0.18
$V_{i(}$	-0.25	0.08	0.09	-0.10	-0.04	0.18	1.00

Animal F13-E-2

	β	δ	α	p	c	σ	$V_{i(}$
β	1.00	-0.35	-0.54	-0.04	0.10	-0.24	-0.21
δ	-0.35	1.00	0.35	0.10	-0.31	0.04	0.08
α	-0.54	0.35	1.00	-0.18	0.07	0.20	0.05
p	-0.04	0.10	-0.18	1.00	0.13	-0.01	-0.14
c	0.10	-0.31	0.07	0.13	1.00	-0.38	-0.05
σ	-0.24	0.04	0.20	-0.01	-0.38	1.00	0.00
$V_{i(}$	-0.21	0.08	0.05	-0.14	-0.05	0.00	1.00

Animal F13-E-3

	β	δ	α	p	c	σ	$V_{i(}$
β	1.00	-0.23	-0.40	-0.01	0.39	-0.31	-0.12
δ	-0.23	1.00	0.16	0.10	-0.39	0.02	-0.11
α	-0.40	0.16	1.00	-0.06	0.42	0.06	0.01
p	-0.01	0.10	-0.06	1.00	-0.06	0.21	-0.46
c	0.39	-0.39	0.42	-0.06	1.00	-0.28	0.04
σ	-0.31	0.02	0.06	0.21	-0.28	1.00	-0.11
$V_{i(}$	-0.12	-0.11	0.01	-0.46	0.04	-0.11	1.00

Animal CTan-H-1

	β	δ	α	p	c	σ	$V_{i(}$
β	1.00	-0.13	-0.40	0.03	0.25	-0.32	-0.03
δ	-0.13	1.00	0.30	-0.03	-0.18	-0.06	0.17
α	-0.40	0.30	1.00	-0.13	0.34	0.03	0.05
p	0.03	-0.03	-0.13	1.00	0.00	-0.07	-0.04
c	0.25	-0.18	0.34	0.00	1.00	-0.25	0.12

σ	-0.32	-0.06	0.03	-0.07	-0.25	1.00	-0.06
$V_{i(}$	-0.03	0.17	0.05	-0.04	0.12	-0.06	1.00

Animal CTan-H-2

	β	δ	α	p	c	σ	$V_{i(}$
β	1.00	-0.05	-0.41	-0.30	0.28	-0.47	-0.30
δ	-0.05	1.00	0.01	-0.05	-0.30	0.19	0.02
α	-0.41	0.01	1.00	-0.06	0.17	0.26	0.10
p	-0.30	-0.05	-0.06	1.00	0.14	-0.14	0.12
c	0.28	-0.30	0.17	0.14	1.00	-0.53	-0.08
σ	-0.47	0.19	0.26	-0.14	-0.53	1.00	0.14
$V_{i(}$	-0.30	0.02	0.10	0.12	-0.08	0.14	1.00

Animal CTan-H-3

	β	δ	α	p	c	σ	$V_{i(}$
β	1.00	-0.07	-0.39	-0.27	0.45	-0.43	-0.26
δ	-0.07	1.00	0.11	-0.12	-0.46	0.34	0.21
α	-0.39	0.11	1.00	0.00	0.16	0.18	0.06
p	-0.27	-0.12	0.00	1.00	-0.09	0.03	-0.04
c	0.45	-0.46	0.16	-0.09	1.00	-0.49	-0.22
σ	-0.43	0.34	0.18	0.03	-0.49	1.00	0.08
$V_{i(}$	-0.26	0.21	0.06	-0.04	-0.22	0.08	1.00
